# Perturbed epigenetic transcriptional regulation in AML with IDH mutations causes increased susceptibility to NK cells

**DOI:** 10.1038/s41375-023-01972-3

**Published:** 2023-07-26

**Authors:** Anna Palau, Filip Segerberg, Michael Lidschreiber, Katja Lidschreiber, Aonghus J. Naughton, Maria Needhamsen, Lisa Anna Jung, Maja Jagodic, Patrick Cramer, Sören Lehmann, Mattias Carlsten, Andreas Lennartsson

**Affiliations:** 1 Department of Biosciences and Nutrition, Karolinska Institutet, Stockholm, Sweden; 2 Center for Hematology and Regenerative Medicine, Department of Medicine Huddinge, Karolinska Institutet, Stockholm, Sweden; 3 Department of Molecular Biology, Max Planck Institute for Multidisciplinary Sciences, Göttingen, Germany; 4 Department of Clinical Neuroscience, Center for Molecular Medicine, Karolinska Institutet, Solna, Sweden; 5 Hematology Centre, Karolinska University Hospital, Stockholm, Sweden; 6 Hematology Unit, Department of Medical Sciences, Uppsala University, Uppsala, Sweden; 7 Center for Cell Therapy and Allogeneic Stem Cell Transplantation, Karolinska Comprehensive Cancer Center, Karolinska University Hospital, Stockholm, Sweden

**Keywords:** Acute myeloid leukaemia, Preclinical research

## Abstract

Isocitrate dehydrogenase (IDH) mutations are found in 20% of acute myeloid leukemia (AML) patients. However, only 30–40% of the patients respond to IDH inhibitors (IDHi). We aimed to identify a molecular vulnerability to tailor novel therapies for AML patients with IDH mutations. We characterized the transcriptional and epigenetic landscape with the IDH2i AG-221, using an IDH2 mutated AML cell line model and AML patient cohorts, and discovered a perturbed transcriptional regulatory network involving myeloid transcription factors that were partly restored after AG-221 treatment. In addition, hypermethylation of the HLA cluster caused a down-regulation of HLA class I genes, triggering an enhanced natural killer (NK) cell activation and an increased susceptibility to NK cell-mediated responses. Finally, analyses of DNA methylation data from IDHi-treated patients showed that non-responders still harbored hypermethylation in HLA class I genes. In conclusion, this study provides new insights suggesting that IDH mutated AML is particularly sensitive to NK cell-based personalized immunotherapy.

## Introduction

Acute myeloid leukemia (AML) is a poor prognosis disease with recurrent genetic alterations, including somatic mutations and chromosomal alterations defining clinical subtypes [1]. Mutations in isocitrate dehydrogenase (*IDH*) 1 or 2 are present in approximately 20% of AML patients. Both IDH1 and IDH2 catalyze the production of α-ketoglutarate (α-KG). However, when mutated the oncometabolite (R) enantiomer of 2-hydroxyglutarate (R)-2HG or 2-HG is produced instead, which competitively inhibits α-KG-dependent enzymes, including members of the ten-eleven-translocation (TET) family of 5-methylcytosine hydroxylases, lysine histone demethylases, and prolyl hydroxylases [2, 3]. TET2 regulates the transition from DNA methylation (5mC) to hydroxymethylation (5hmC). As a consequence of such TET2 inhibition, AML blast cells that harbor IDH1 or IDH2 mutations display a global loss of 5hmC and a differentiation arrest that contributes to leukemogenesis [4, 5].

Inhibitors of mutated IDH1 and IDH2 have recently been developed for AML treatment. Enasidenib (AG-221) is an inhibitor of mutated IDH2, which induces differentiation [6–8]. AG-221 is efficient as a single-drug treatment with response rates of 30–40% [9–11]. Recently, Wang et al. showed that gene expression signatures associated with stemness are associated with primary resistance to IDHi, whereas the selection of resistant mutations plays a role in acquired resistance to the drugs [12]. In addition, most patients relapse, and the increased overall survival is less than a year [8, 9]. Thus, despite the new treatment strategies that AG-221 offers, the efficacy needs to be improved and additional treatment modalities are required.

Natural Killer (NK) cells are immune cells with an innate ability to recognize and kill malignantly transformed cells, including AML [13, 14]. Upon activation, NK cells degranulate to release cytotoxic molecules such as perforin and granzyme B, which can directly induce target cell death. Furthermore, activated NK cells produce pro-inflammatory cytokines such as Interferon-γ (IFN-γ) and Tumor Necrosis Factor-α (TNF-α) that stimulate other parts of the immune system [15, 16]. Activation and subsequent response of an NK cell towards a target cell is regulated by an intricate balance of signals from both activating and inhibitory receptors. Activating receptors recognize stress-induced ligands, whereas inhibitory receptors mainly recognize different subtypes of HLA class I molecules present on the target cell surface. Down-regulation or loss of HLA class I expression is commonly observed in cancer and can result in strong NK cell activation and subsequent effector responses, referred to as 'missing-self' recognition [17, 18].

Previous studies have shown that NK cells can prevent relapse and induce remission in patients with poor prognosis AML or MDS [19, 20]. However, only subgroups of patients respond to NK cell therapy [21]. Thus, knowledge of how to select suitable patients is essential for successful treatment.

In this study, we characterized transcriptional and epigenetic alterations resulting from IDH mutations and investigated the effects of IDHi. Moreover, we demonstrate that IDH mutated AML cells trigger elevated NK cell-mediated responses. Our data suggest that adoptive NK cell-based immunotherapy can be a treatment option for IDH mutated AML patients.

## Materials and methods

### Cell culture

The AML TF-1 cell lines overexpressing mutated IDH2R140Q or IDH2WT were kindly provided by Agios Pharmaceuticals [6]. PBMCs were obtained from healthy donors in accordance with existing ethical permits (2006/229-31/3) using high-density gradient centrifugation. Upon thawing, NK cells were purified from PBMCs by magnet-assisted negative depletion using an NK cell isolation kit (Miltenyi). Before use, PBMCs and NK cells were overnight cultured in RPMI 1640 medium (Gibco) supplemented with 10% FBS (Gibco) and 1000 IU/mL IL-2 (Peprotech) at 37 °C in 5% CO_2_. For more information see Supplementary Materials and Methods.

### TT-seq, RNA-seq, and DNA methylation and hydroxymethylation analysis

TT-seq experiments were performed in biological duplicates (Spearman correlations between replicates >0.98). A complete TT-seq step-by-step protocol can be found in the protocols.io repository [22]. Reads that did not map to the ribosomal DNA (rDNA) were aligned to the GRCh38 genome assembly (Human Genome Reference Consortium) using STAR 2.6.0c [23]. Annotation of enhancer RNAs (eRNAs) was done as described [24] with few modifications, see Supplementary Materials and Methods.

DNA methylation and hydroxymethylation assays were performed using an Infinium EPIC array (Illumina) at NXT-Dx (Diagenode). Genomic DNA was subjected to bisulfite (BS)-treatment and oxidative BS (oxBS)-treatment using the EZ-96 DNA Methylation Kit (Zymo Research) according to Illumina’s recommended deamination protocol. For more information see Supplementary Materials and Methods.

### Phenotyping by flow cytometry

To evaluate HLA class I surface protein expression and intensity, tumor cells were labeled with anti-human fluorescently-conjugated antibodies. Zombie NIR Fixable Viability kit (Biolegend) was used to discriminate between live and dead cells. All cells were acquired on an LSR II Fortessa instrument (BD Biosciences).

### Degranulation, cytokine production, and cytotoxicity assay

Overnight IL-2 activated PBMCs were co-cultured with target cells at an effector to target (E:T) cell ratio of 10:1. To measure degranulation, anti-CD107a-BV785 (LAMP-1) (Biolegend) was added before initiating the co-culture experiment. The NK cell cytotoxicity assay was performed in a similar way to what we have previously described [25]. For more information see Supplementary Materials and Methods.

## Results

### AG-221 treatment increases 5hmC at enhancer sites in IDH2R140Q AML

We used the AML cell line TF-1, overexpressing mutant R140Q or wild type (WT) IDH2 as a model system [6, 26, 27]. TF-1 IDH2R140Q cells produced 2-HG (Fig. S1A), demonstrating that our model resembles primary AML cells with IDH mutations. Similar to AML patients with IDH mutations [4, 5], the TF-1 IDH2R140Q cells displayed a hypermethylated DNA profile, consisting of 141406 hypermethylated sites and 65615 hypomethylated sites (Fig. S1B). Consistent with the TET2 inhibitory function of IDH2R140Q, the 5hmC levels were reduced in IDH2R140Q (Fig. S1C), and three times as many sites lost 5hmC (2156 sites) compared to gained 5hmC (664 sites) (Fig. S1D). Analyzing the genomic location of the lost and gained 5hmC sites, we found that both lost and gained 5hmC sites were enriched in enhancers and gene bodies and depleted in promoters (Fig. S1E). Moreover, the de-regulated 5hmC sites were enriched in distal (4kbp) regions from CpG islands, called Open seas, whereas CpG islands and their vicinity (CpG shores, up to 2kbp) were depleted for changed 5hmC (Figure S1F). The changes for 5hmC and 5mC displayed an anti-correlative pattern (*r* = −0.737) (Fig. S1G). This is in agreement with previous studies showing that de-regulated 5mC and 5hmC in AML blasts with IDH mutations are enriched for enhancer regions [26, 28].

**Fig. 1:**
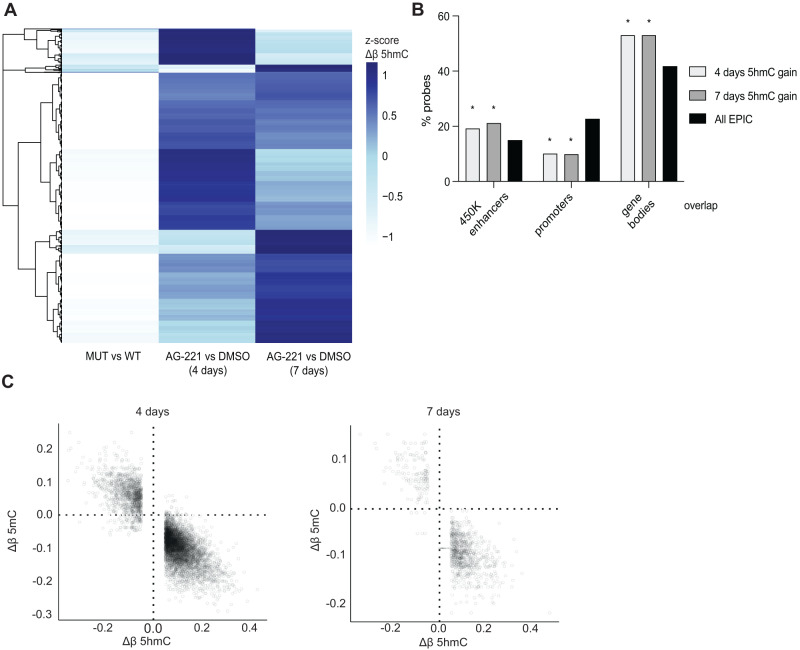
Reduced hydroxymethylation in TF-1 IDH2R140Q is reversed after AG-221 treatment. **A** Heatmap showing z-scores of Δβ 5hmC values of the 2156 CpG sites which lose 5hmC in TF-1 IDH2R140Q mutant vs TF-1 IDH2WT cells in steady-state and AG-221 treatment (4 and 7 days). **B** Genomic location in 450 K enhancers, promoters or gene bodies (based on UCSC RefGene group; not annotated, 3’UTR and 5’UTR are not shown) of the hyperhydroxymethylated CpG probes (Δβ 5hmC > 0.05) at 4 and 7 days of AG-221 treatment in TF-1 IDH2R140Q mutant cells, comparing to location of all CpG probes in the EPIC array. Enrichment was calculated using Fisher exact *t*-test. *p-value < 0.01. **C** Dotplots of 5hmC deltabeta value (Δβ-value 5hmC) of differentially hydroxymethylated CpG probes (absolute(Δβ-value 5hmC) > 0.05) in relation to their 5mC deltabeta value (Δβ-value 5mC) between TF-1 IDH2R140Q cells treated with AG-221 for 4 days (Pearson correlation coefficient of −0.848) and 7 days (Pearson correlation coefficient of −0.815) and DMSO-treated control cells.

To analyze the molecular drug response, TF-1 IDH2R140Q cells were incubated with AG-221. A reduction of the oncometabolite 2-HG levels and an increased 5hmC were detected after 4 and 7 days of AG-221 treatment (Fig. S1H, I). The 2156 sites that had decreased 5hmC levels in TF-1 IDH2R140Q re-gained their 5hmC levels after 4 and 7 days of AG-221 incubation (Fig. 1A and Fig. S1J). Similar to the steady state (Fig. S1E), enhancers and gene bodies were enriched for sites with gained 5hmC, while promoters were depleted (Fig. 1B). Accordingly, CpG islands were depleted and Open Seas were enriched for gained 5hmC (Fig. S1K). The sites that gained 5hmC after AG-221 treatment displayed a corresponding reduction in 5mC (*r* = −0.848 and *r* = −0.815, at 4 and 7 days) (Fig. 1C). In addition, the 664 sites that gained 5hmC levels in TF-1 IDH2R140Q compared to TF-1 IDH2WT displayed a decreased 5hmC after 4 and 7 days of AG-221 treatment (Fig. S1L). Together these results suggest that AG-221 restored the 5hmC levels at both gained and lost sites in TF-1 IDH2R140Q cells, including enhancers.

### De-regulated promoter and enhancer activity in AML with IDH2 mutation

**Fig. 2:**
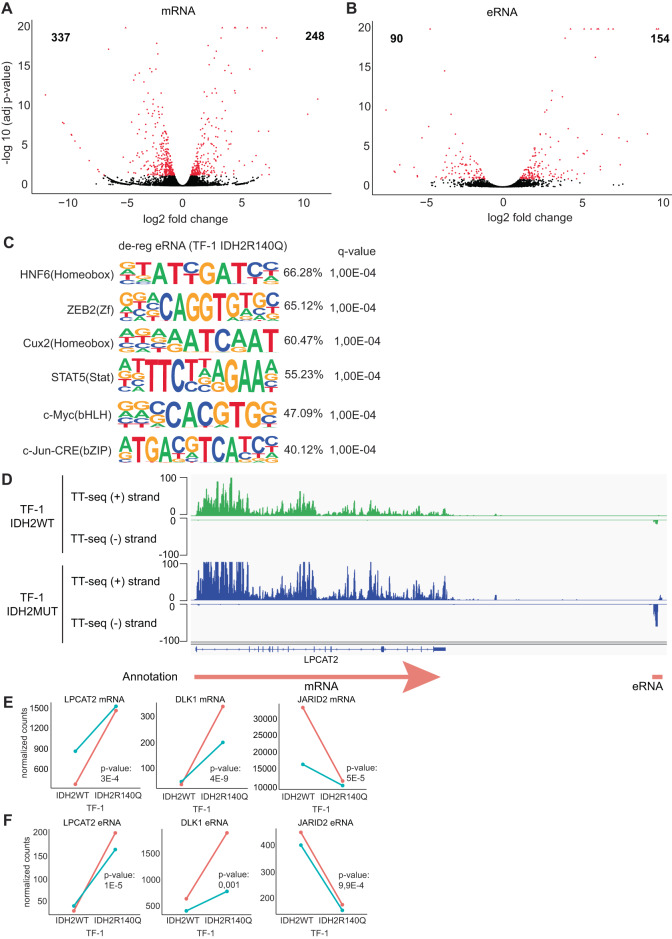
TF-1 IDH2WT and IDH2R140Q cells show different expression profiles. **A** Volcano plot of mRNA expression between TF-1 IDH2R140Q and TF-1 IDH2WT cells in total extracted RNA (*n* = 2). Plotted along the x-axis is the mean of log2 fold-change, along the y-axis the negative logarithm of the adjusted *p*-values. Red denotes the 248 up-regulated protein-coding genes and the 337 down-regulated protein-coding genes in the IDH2 mutant versus WT cells (*p*-adj value < 0.05). Black denotes non-significantly differentially expressed protein-coding genes. **B** Volcano plot of enhancer RNA (eRNA) expression between TF-1 IDH2R140Q and TF-1 IDH2WT cells in labeled extracted RNA (*n* = 2). Red denotes the 154 up-regulated eRNAs and the 90 down-regulated eRNAs in the IDH2 mutant versus WT cells (*p*-adj value < 0.1). Black denotes non-significantly differentially expressed eRNAs. **C** Motif analysis in eRNAs of up- and down-regulated eRNAs in the TF-1 IDH2R140Q versus IDH2WT cells. Percentage indicates the % of Targets Sequences with Motif. **D** Exemplary IGV genome browser view of TT-seq coverage with TF-1 IDH2R140 tracks in blue and TF-1IDH2WT tracks in green and transcript annotation at the LPCAT2 locus (hg38; chr16: 55,507,632- 55,654,834). Due to the high expression of LPCAT2, the TT-seq coverage is cut at 150 to allow for better visualization of the surrounding eRNA signal. **E** LPCAT, DLK1 and JARID2 normalized mRNA counts in IDH2WT and IDH2R140 mutant (IDH2MUT) cells. **F** LPCAT, DLK1(eRNA.inter.merged.1962) and JARID2 normalized eRNA counts in IDH2WT and IDH2R140 mutant (IDH2MUT) cells. The two replicates are indicated with different color.

To investigate changes in gene expression, we performed RNA-seq in TF-1 IDH2R140Q and TF-1 IDH2WT cells. In agreement with the observed hypermethylation profile (Fig. S1B), a higher proportion of the de-regulated genes were down-regulated compared to the up-regulated (Fig. 2A and Table S1). Genes involved in differentiation, development, myeloid cell activation, and proliferation were down-regulated (Fig. S2A). Gene set enrichment analysis (GSEA) showed that several immune response gene sets were down-regulated, such as allograph rejection (Fig. S2B). Furthermore, 90 long non-coding RNAs (lncRNAs) were de-regulated by the IDH2R140Q mutation (Fig. S2C).

Since changes in 5hmC were particularly abundant in enhancers (Fig. 1B), we further analyzed enhancer RNAs (eRNAs) to annotate transcriptionally active enhancers as a proxy for enhancer activity [29–32] (Fig. S2D). We used transient transcriptome sequencing (TT-seq) to analyze newly synthesized eRNAs with high sensitivity [24, 33]. We annotated 4998 putative eRNAs in TF-1 IDH2R140Q and TF-1 IDH2WT cells. Differential expression analysis detected 244 de-regulated eRNAs in TF-1 IDH2R140Q (Fig. 2B). Motif search analysis showed that the de-regulated enhancers were enriched in binding sites for Homeobox transcription factors, c-Myc, ZEB2, STAT5 and the AP-1 factor c-Jun (Fig. 2C). AP-1 has previously been shown to be a key transcription factor for enhancer regulation [29]. Transcriptional co-regulation between enhancers and promoters was suggested by the transcription factor STAT5, whose binding sites were enriched in the de-regulated enhancers and GSEA showed that expression of STAT5 target genes was also altered TF-1 IDH2R140Q (Fig. 2C and Fig. S2B). To further link enhancer activity to the activation of target gene transcription, differentially transcribed enhancers were associated with co-regulated promoter transcription within a +/− 500 kbp window. Using this strategy we identified 48 putative co-regulated enhancer-promoter pairs (Table S2). For example, the expression of Lysophosphatidylcholine acyltransferase 2 (LPCAT2) which is involved in the generation of Platelet-activating factor (PAF), was elevated in TF-1 IDH2R140Q, along with increased activity at a putative enhancer (Fig. 2D–F). Also, the mRNA expression of DLK1 that has been implicated in myelodysplastic syndrome [34] and the Polycomb repressive complex 2 (PRC2) member JARID2 were potentially de-regulated by nearby putative enhancers (Fig. 2E, F and Fig. S2E). These results suggest that transcriptional de-regulation at a set of promoters is linked to perturbed enhancer activity in TF-1 IDH2R140Q cells.

### AG-221 treatment induces a myeloid transcription factor driven response at promoters and enhancers

**Fig. 3:**
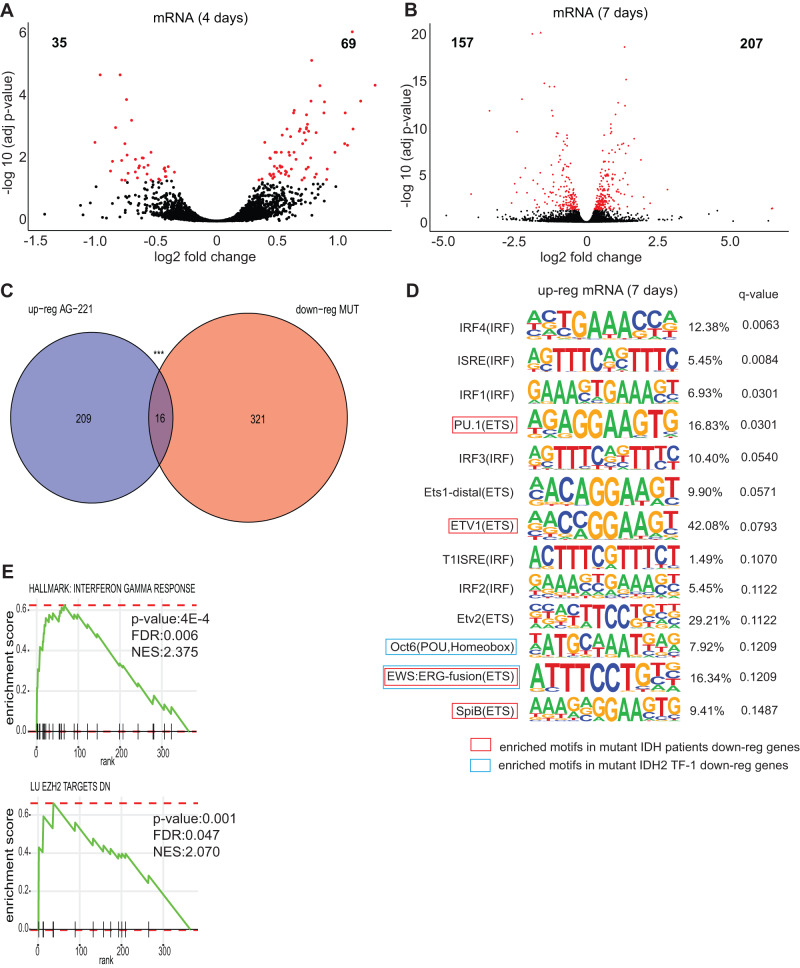
Expression profile of TF-1 IDH2R140Q mutant cells treated with AG-221. **A** Volcano plot of mRNA expression between TF-1 IDH2R140Q AG-221/DMSO treated cells during 4 days in total extracted RNA (*n* = 2). Plotted along the x-axis is the mean of log2 fold-change, and along the y-axis is the negative logarithm of the adjusted p-values. Red denotes the 69 up-regulated protein-coding genes and the 35 (4 days) down-regulated protein-coding genes in the AG-221 versus DMSO treated TF-1 IDH2R140Q mutant cells (*p*-adj value < 0.05). Black denotes non-significantly differentially expressed protein-coding genes. **B** Volcano plot of mRNA expression between TF-1 IDH2R140Q AG-221/DMSO treated cells during 7 days in total extracted RNA (*n* = 2). Plotted along the x-axis is the mean of log2 fold-change, and along the y-axis is the negative logarithm of the adjusted *p*-values. Red denotes the 207 up-regulated protein-coding genes and the 157 down-regulated protein-coding genes in the AG-221 versus DMSO treated TF-1 IDH2R140Q mutant cells (*p*-adj value < 0.05). Black denotes non-significantly differentially expressed protein-coding genes. **C** Venn diagram showing the overlap of down-regulated genes in TF-1 IDH2R140Q compared to IDH2WT with up-regulated genes at 4 or 7 days of AG-221 treatment in TF-1 IDH2R140Q cells (***p-value < 0.001; hypergeometric test *P* = 9.95e-06). **D** Motif analysis in promoters of up-regulated protein-coding genes in TF-1 IDH2R140Q cells treated with AG-221 for 7 days. Percentage indicates the % of Targets Sequences with Motif. Common motifs with down-regulated genes in IDH mutated patients compared to IDH WT (ClinSeq AML patients cohort) are framed in red and common motifs with down-regulated genes in TF-1 IDH2R140Q compared to IDH2WT are framed in blue. **E** Gene set enrichment analysis (GSEA) plots comparing gene expression profiles between TF-1 IDH2R140Q treated with AG-221 or DMSO for 7 days. Normalized enrichment score (NES).

The transcriptional response after 4 and 7 days of AG-221 treatment in TF-1 IDH2R140Q cells was analyzed by RNA-seq and TT-seq. After 4 days, almost twice as many protein-coding genes were up-regulated compared to down-regulated (Fig. 3A and Table S3). Furthermore, after 7 days of AG-221 treatment, more of the affected genes were up-regulated (Fig. 3B and Table S4). Most of the up-regulated genes after 4 days of treatment remained up-regulated after 7 days of treatment (Fig. S3A). Even though most of the down-regulated 337 genes in TF-1 IDH2R140Q cells are not re-activated by AG-221 treatment, a significant gene set (16 genes) became up-regulated after 7 days AG-221 incubation (Fig. 3C). The 5mC levels in CpG sites located in the up-regulated 16 promoters were reversed after treatment (Fig. S3B) but without statistically significant correlation between changes in 5hmC and changes in gene transcription. As previously described, the relation between 5hmC and gene expression is complex and dependent on genomic location and cell type [35]. Transcription factor motif analysis indicated that the gene transcription activation after 7 days of treatment was regulated by interferon regulatory factors (IRF1–4, ISRE), ETS factors (PU.1, ETS1, ETV1–2, EWS, SPIB) and the homeobox factor OCT6 (Fig. 3D). Protein interaction network analysis showed that most of these transcription factors interact with each other network (Fig. S3C). Several of these motifs (PU.1, ETV1, EWS:ERG-fusion and SPIB) were also enriched in down-regulated gene promoters in IDH mutated patients, suggesting a re-activation of the transcription factor network after AG-221 incubation (Table S5 and marked in red in Fig. 3D). Moreover, some of the AG-221 re-activated transcription factors, Oct6 and EWS:ERG-fusion motifs were also enriched in the repressed promoters in TF-1 IDH2R140Q cells (Table S6 and marked in blue in Fig. 3D). Some of these transcription factors are involved in myeloid development [36]. GSEA and GO-analysis of the up-regulated genes after 7 days of AG-221 treatment confirmed the role of interferon gamma response and induced myeloid differentiation (Figs. 3E, S3D–F). Several AG-221 induced gene sets were involved in EZH2 (PRC2) targets (Fig. 3E). Polycomb repressive complex regulates and represses genes that are involved in myeloid development [37]. Up-regulation of Polycomb targets, therefore, indicated induction of myeloid differentiation.

**Fig. 4:**
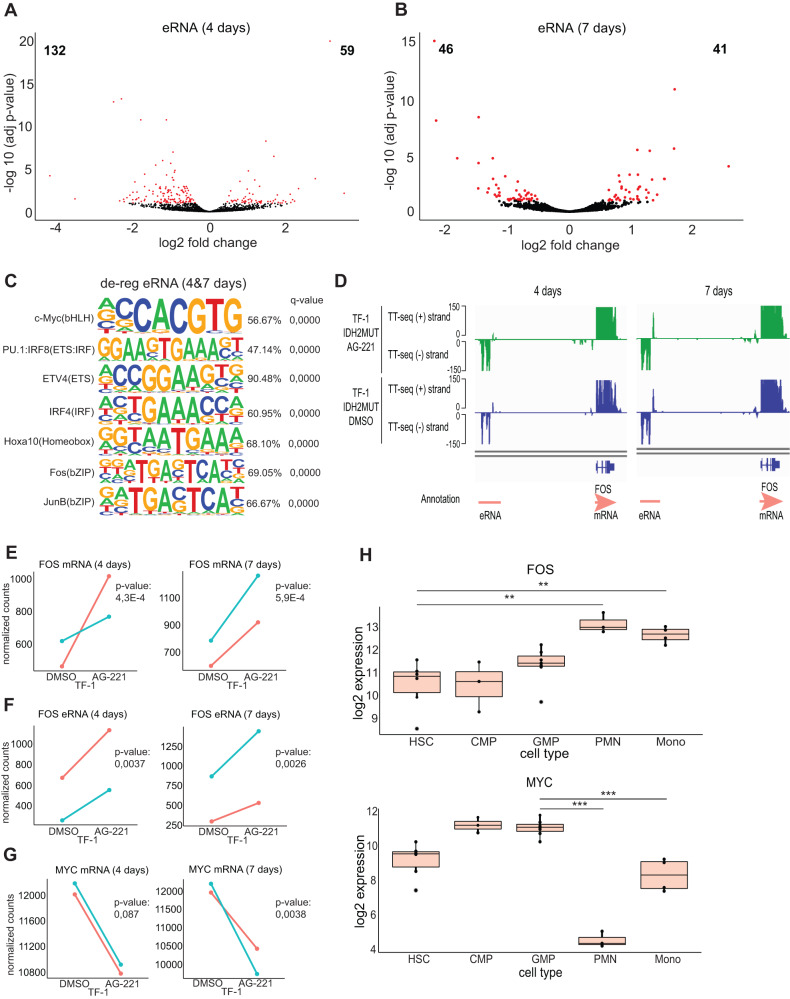
Enhancer RNA transcription in TF-1 IDH2R140Q mutant cells treated with AG-221. **A** Volcano plot of enhancer RNA (eRNA) expression between TF-1 IDH2R140Q treated with AG-221 or DMSO for 4 days in labeled extracted RNA (*n* = 2). Red denotes the 59 up-regulated eRNAs and the 132 down-regulated eRNAs in the IDH2 mutant AG-221-treated versus DMSO-treated cells (p-adj value < 0.1). Black denotes non-significantly differentially expressed eRNAs. **B** Volcano plot of enhancer RNA (eRNA) expression between TF-1 IDH2R140Q treated with AG-221 or DMSO for 7 days in labeled extracted RNA (*n* = 2). Red denotes the 41 up-regulated eRNAs and the 46 down-regulated eRNAs in the IDH2 mutant AG-221-treated versus DMSO-treated cells (*p*-adj value < 0.1). Black denotes non-significantly differentially expressed eRNAs. **C** Motif analysis in eRNAs of de-regulated eRNAs in the TF-1 IDH2R140Q AG-221-treated versus DMSO-treated cells for 4 and 7 days. Percentage indicates the % of Targets Sequences with Motif. **D** Exemplary IGV genome browser view of TT-seq coverage with TF-1 IDH2R140 tracks treated with AG-221 in green or DMSO in blue for 4 or 7 days and transcript annotation at the FOS locus (hg38; chr14: 75,256,352-75,284,688). Due to the high expression of FOS, the TT-seq coverage is cut at 150 to allow for better visualization of the surrounding eRNA signal. **E** FOS normalized mRNA counts in IDH2R140 mutant (IDH2MUT) cells treated with DMSO or AG-221 for 4 and 7 days. **F** FOS normalized eRNA counts in IDH2R140 mutant (IDH2MUT) cells treated with DMSO or AG-221 for 4 and 7 days. **G** MYC normalized mRNA counts in IDH2R140 mutant (IDH2MUT) cells treated with DMSO or AG-221 for 4 and 7 days. The two replicates are indicated with different colors. **H** Boxplot representing logarithmic RNA expression of FOS and MYC genes during normal granulocytic/monocytic differentiation [55]. Hematopoietic stem cell (HSC), common myeloid progenitor (CMP), granulocyte-macrophage progenitor (GMP), polymorphonuclear (PMN) mature granulocyte and monocyte (mono). **p-value < 0.01; ***p-value < 0.001.

Differential expression analysis of eRNAs detected 59 and 132 significantly up- and down-regulated eRNAs after 4 days of treatment and 41 and 46 significantly up- and down-regulated eRNAs after 7 days (Fig. 4A, B). Similar to gene promoters, the affected enhancer regions were enriched for ETS (e.g PU.1) and IRF factors, and also for Myc and the AP-1 factors Fos and JunB (Fig. 4C), consistent with the observed enrichment for Myc and Jun binding sites in IDH2R140Q de-regulated enhancers (Fig. 2C). Furthermore, the expression levels of such transcription factors were also significantly affected by AG-221 treatment. The expression levels of FOS, JUN, and ZEB2 were decreased by IDH2R140Q (Fig. S4A) but reverted after AG-221 treatment (Fig. 4E and Figure S4B). In contrast, the expression of the MYC oncogene was increased in IDH2R140Q cells (Fig. S4C) and then decreased after AG-221 (Fig. 4G). Also, an enhancer upstream of the FOS gene became activated that may regulate FOS expression (Fig. 4D and F). Fos/Jun transcription factor complexes have been shown to positively regulate myeloid differentiation [38, 39]. This was in line with the observed up-regulation of FOS that occurred during myeloid differentiation, while MYC expression decreased with differentiation (Fig. 4H).

### Down-regulation of the HLA cluster in AML with IDH mutations increases sensitivity to NK cell-mediated responses and killing

**Fig. 5:**
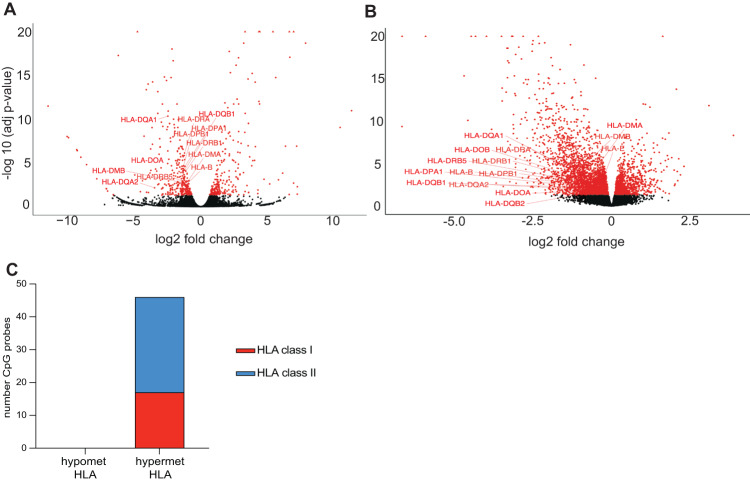
HLA down-regulation in IDH mutated AML. **A** Volcano plot of mRNA expression between TF-1 IDH2R140Q and TF-1 IDH2WT cells in total extracted RNA (*n* = 2). Plotted along the x-axis is the mean of log2 fold-change, and along the y-axis is the negative logarithm of the adjusted p-values. Significantly down-regulated HLA genes in the IDH2 mutant versus WT cells (p-adj value < 0.05) are highlighted. **B** Volcano plot of mRNA expression between AML patients within the ClinSeq cohort with IDH1/2 mutations (*n* = 87) and IDH WT patients (*n* = 234). Plotted along the x-axis is the mean of log2 fold-change, and along the y-axis is the negative logarithm of the adjusted *p*-values. Significantly down-regulated HLA genes in the IDH mutant versus WT cells (*p*-adj value < 0.05) are highlighted. **C** Barplot indicating a number of hypermethylated (*n* = 46) or hypomethylated (*n* = 0) CpG probes in HLA genes comparing IDH1/2 mutated AML patients (with DNMT3AWT) (*n* = 26) versus IDHWT (with DNMT3AWT) (*n* = 69) in BS samples. The class of HLA (I or II) is shown.

Both the down-regulated GO-term “immune system process” and the GSEA “Hallmark allograft rejection” (Fig. S2A, B) included HLA genes. Therefore, we analyzed the expression of HLA genes in TF-1 IDH2R140Q and found that HLA genes were significantly enriched among the down-regulated genes (*P* = 4.434e-15, Fisher’s exact test). Further analysis of the IDH2R140Q transcriptional consequence revealed a strong down-regulation of genes in the HLA class I and II clusters in TF-1 IDH2R140Q (Fig. 5A, Table S7). We confirmed this down-regulation of HLA class I and II clusters in a large AML cohort [40] (Fig. 5B, Table S8). The AML cohort displayed a strong hypermethylation profile in the IDH mutated patients that include the HLA clusters (Fig. S5A). The hypermethylated HLA genes corresponded to both HLA class I and class II (Fig. 5C), showing strong hypermethylation both in promoters and gene bodies (Figure S5B). Additionally, in the TF-1 IDH2R140Q cell line, hypermethylation of HLA gene promoters could be observed (Fig. S5C). Like the patient data, we also found HLA class I and class II genes in hypermethylated HLA probes in the TF-1 mutant cells (Fig. S5D).

**Fig. 6:**
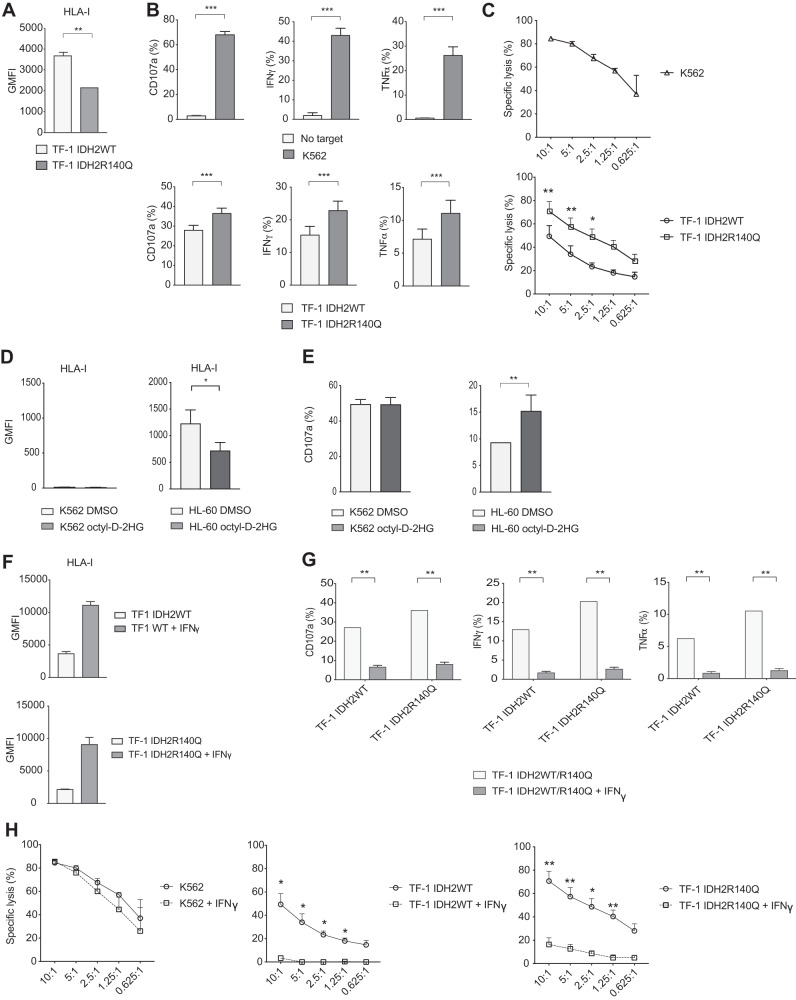
Functional response of NK cells towards TF-1 IDH2R140Q compared to TF-1 IDH2WT cells. **A** Barplot showing geometric mean fluorescence intensity (GMFI) of pan HLA class I molecules for TF-1 IDH2WT and TF-1 IDH2R140Q cell lines from three independent staining’s (*n* = 3). **B** Barplots indicating the percent of NK cells within the PBMC compartment that stained positively for CD107a, IFN-γ, and TNF-α expression against denoted target cells. NK cells were identified as being CD56 positive and CD3 negative. Data was obtained from 3 independent experiments (*n* = 13). Bars indicate mean. Error bars indicate SEM (**C**) Specific lysis of denoted target cells determined by a Calcein-AM based cytotoxicity assay. The specific effector to target (E:T) ratios are specified on the x-axis. Each point at each indicated E:T ratio represents the mean of 3 donors (*n* = 3) and each was performed in triplicates. **D** Barplot showing geometric mean fluorescence intensity (GMFI) of pan HLA class I molecules for HL-60 and K562 cell lines treated with DMSO or octyl-D-2HG for 14 days (*n* = 3). **E** Barplots indicating the percent of NK cells within the PBMC compartment that stained positively for CD107a against denoted target cells treated with DMSO or octyl-D-2HG for 14 days. NK cells were identified as being CD56 positive and CD3 negative (*n* = 6). **F** Barplots showing geometric mean fluorescence intensity (GMFI) of pan HLA class I molecules for TF-1 IDH2WT and TF-1 IDH2R140Q cell lines stimulated with or without 10 ng/mL of IFN-γ Recombinant Human Protein for 48 h prior to staining. Results were obtained from two independent staining’s (*n* = 2). **G** Barplots indicating the percent of NK cells within the PBMC compartment that stained positively for CD107a, IFN-γ and TNF-α expression against denoted target cells that had either been or not been, stimulated with above mentioned dose of IFN-γ for 48 h prior to the assays. NK cells were identified as being CD56 positive and CD3 negative. Data was obtained from two independent experiments (*n* = 10). Bars indicate mean. Error bars indicate SEM. **H** Specific lysis of denoted target cells that had either been or not been, stimulated with above mentioned dose of IFN-γ for 48 h prior to the assay determined by a Calcein-AM based cytotoxicity assay. The specific effector to target (E:T) ratios are specified on the x-axis. Each point at each indicated E:T ratio represents the mean of 3 donors (*n* = 3) and each was performed in triplicates. Error bars represent SEM. Paired or unpaired *t*-tests were performed for all paired or unpaired analysis in this figure respectively. When no statistical significance is noted, it was either not possible to perform the test (due to low sample size), or because the result was non-significant. *p-value < 0.5; **p-value < 0.01; ***p-value < 0.001.

The decreased HLA mRNA expression caused a reduction of HLA class I molecules on the cell surface in TF-1 IDHR140Q (Fig. 6A). When staining for specific HLA class I molecules, a significantly decreased expression was observed for HLA-Bw4, HLA-Bw6, and HLA-C, but not for HLA-E which together with HLA-Bw4 was expressed at very low baseline levels in the TF1 IDH2WT cell line (Fig. S6A and S6B). HLA-A3 did not appear to be expressed at all. As HLA class I molecules regulate NK cells, we further analyzed whether TF-1 IDHR140Q cells would trigger a stronger NK cell response compared to TF-1 IDH2WT cells. TF-1 mutant and WT cells were co-cultured overnight with IL-2 activated peripheral blood mononuclear cells (PBMCs) from healthy donors. Degranulation, measured by CD107a expression, and production of the pro-inflammatory cytokines IFN-γ and TNF-α, among constituent NK cells were quantified. As a positive control, the HLA class I deficient K562 cell line was used. TF-1 IDH2R140Q cells triggered enhanced NK cell degranulation compared to TF-1 IDH2WT cells. Similarly, we could detect a significant increase in IFN-γ and TNF-α production (Figs. 6B and S6C). The observed increased sensitivity was confirmed with a cytotoxicity assay, where overnight IL-2 activated NK cells more efficiently lysed IDH2R140Q mutated cells compared to IDH2WT cells at different E:T ratios (Fig. 6C). To link our observations to specific inhibitory receptor – ligand interactions, we further looked at the effector responses in different NK cell subsets. Using a Boolean gating strategy, where subsets were identified based on the expression of a single HLA class I binding inhibitory receptor (single positive: SP), or by the absence of all inhibitory HLA class I binding receptors that we stained for (iNKR^−^), we observed an increased degranulation and cytokine production in all investigated subsets (Fig. S6D). Because TF-1 cells expressed HLA class I molecules confined to the HLA-C1 group, while lacking the HLA-C2 variant, we performed a more detailed sub analysis for the KIRD2DL1/DS1 and the KIR2DL2/DS2/DL3 SP subsets together with the inhibitory receptor negative subset. This analysis showed that KIR2DL2/DS2/DL3 SP NK cells that are controlled upon binding of HLA-C1, had a higher degree of response compared to the other NK cell subsets as measured by degranulation and IFN-γ production, but not in TNF-α production, when co-cultured with TF-1 IDH2R140Q instead of TF-1 IDH2WT cells (Fig. S6E).

To investigate the link between 2-HG levels and NK cell sensitivity in a different cell model, we assessed HLA class I levels in HL-60 and K562 leukemia cell lines after treatment with octyl-D-2HG, a membrane-permeant precursor form of 2-HG. After 14 days of treatment, we observed a significant down-regulation of HLA class I in the HL-60 cells and no effect in the HLA class I negative cell line K562 (Fig. 6D), demonstrating a 2-HG dependent repression of HLA class I. Furthermore, HL-60 cells treated with octyl-D-2HG induced an enhanced NK cell degranulation compared to HL-60 vehicle-treated cells while no change in NK cell degranulation was observed in the K562 cells used as a control (Fig. 6E).

To further explore the importance of the HLA class I molecules in regulating NK cell-mediated responses against TF-1 AML cells, HLA class I overexpression was induced by pre-stimulating the cells with IFN-γ 48 h prior to co-culture with NK cells. Indeed, this resulted in an increase in cell surface expression of several HLA class I molecules in both TF-1 IDH2R140Q and WT cells (Fig. 6F, S6F and S6G). To investigate whether this would influence NK cell-mediated responses, we again performed co-cultures with overnight IL-2 activated PBMCs. IFN-γ-stimulated TF-1 cells triggered significantly lower levels of NK cell degranulation and cytokine production compared to the non-stimulated TF-1 counterparts (Fig. 6G and Fig. S6H). Furthermore, this effect was not observed against the HLA class I negative cell line K562 (Fig. S6G, H). Subsequent cytotoxicity assays displayed a reduction of NK cell-mediated killing of the IFN-y treated TF-1 cell lines compared to the untreated counterpart, while the killing of K562 cells was unaffected by the stimulation (Fig. 6H).

### IDHi resistant patients retain a hypermethylated HLA gene profile

**Fig. 7:**
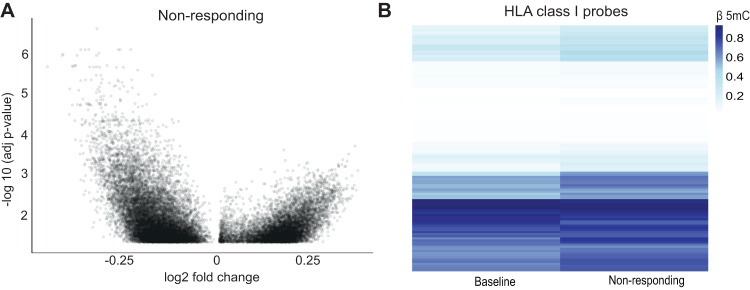
Non-responding AML patients retained hypermethylation in HLA clusters. **A** Volcano plot illustrating differentially total methylated CpG probes (adjusted *p*-value < 0.05) comparing IDH1/2 mutated AML patients at baseline (*n* = 57) versus IDH1/2 mutated AML patients who did not respond to ivosidenib/enasidenib therapy (*n* = 17) in BS samples from the study by Wang and colleagues [12]. 13463 probes were found to be hypomethylated and 8173 hypermethylated in the response samples. **B** Heatmap showing β 5mC values of the 116 CpG sites located in HLA class I genes in IDH1/2 mutated AML patients at baseline (*n* = 57) versus IDH1/2 mutated AML patients who did not respond to ivosidenib/enasidenib therapy (*n* = 17) in BS samples from the study by Wang and colleagues [12].

Recently, Wang and colleagues [12] performed genome-wide DNA methylation analyses to decipher clinical resistance to mutant IDH inhibitors. We analyzed their data to check differentially methylated probes in non-responders after treatment compared to patients at baseline (no treatment) and found 8173 hypermethylated probes and 13463 hypomethylated probes (Fig. 7A), showing increased hypomethylation after treatment with IDH inhibitors in non-responders. However, the hypermethylated HLA class I probes retained their DNA methylation levels after IDHi treatment (Fig. 7B).

## Discussion

In our study, we present a novel concept proposing personalized immunotherapy for AML patients, based on mutational, transcriptional, and epigenetic profiling.

AG-221 treatment has been shown to induce differentiation of IDH2 mutated AML cells [6, 9, 27, 41]. The myeloid differentiation is tightly orchestrated by a network of transcription factors that are essential for complete cell maturation [42]. We show that de-regulated promoters and enhancers in IDH2R140Q AML cells are enriched for specific transcription factors motifs that form an AML-IDH subtype specific network, including RUNX, STAT5, OCT, IRF, PU.1, AP-1 (FOS, JUN), ETS and MYC. Several of those transcription factors are also included in other AML subtype networks, but the combination of them is unique for the AML-IDH subtype [43]. Indeed, inhibition of STAT5 signaling in IDH mutated AML was recently shown to enhance the differentiation response of IDHi treatment [44]. Consistent with our results, Wilson et al. recently showed that motifs for RUNX1, MYC, and PU.1 are enriched in IDH mutation specific hypermethylated regions [26]. Several transcription factors included in the AML-IDH network are either transcriptionally repressed (FOS, ZEB2, JUN) or activated (MYC) in TF-1 IDH2R140Q. AG-221 treatment rewires the transcription factor network by reversing the perturbed transcription factor expression of FOS, JUN, MYC, and ZEB2 and adjusting enhancer and mRNA transcription. We have previously shown that promoters of transcription factors are induced earlier in cellular differentiation and activation than non-transcription factor promoters [45]. Therefore, we speculate that the lack of transcriptional adjustment on non-transcription factor promoters may be due to 7 days of treatment being too short for a normalization of all transcriptional features.

A consequence of the disturbed epigenetic and transcriptional regulation in IDH mutated AML cells is the down-regulation of HLA class I gene expression that regulates NK cell activity [18, 46]. Indeed, IDH2R140Q mutated TF-1 cells triggered stronger NK cell activation, as measured by degranulation and cytokine production, and were more easily killed compared to the TF-1 IDH2WT counterpart. In addition, exogenous octyl-D-2HG treatment of HL-60 cells demonstrated that the 2-HG dependent down-regulation of HLA class I is not TF-1 cell conditional.

The most studied inhibitory HLA class I-binding receptors are the killer cell immunoglobulin-like receptor (KIR) family that binds to classical HLA class I molecules (HLA-A, -B, and -C), the NKG2A receptor that binds to the non-classical HLA class I molecules HLA-E, and the LIR-1 receptor that can bind almost all HLA class I molecules but with lower affinity compared to the aforementioned receptors [47]. As KIR receptors are stochastically expressed by NK cells [48, 49], we performed a boolean gating-based analysis. The aim of this analysis was to more closely investigate the contribution of specific NK cell subsets and potentially link the enhanced response observed against TF-1 cells carrying the IDH2R140Q mutation to specific inhibitory receptor – HLA class I interactions. Interestingly, this analysis revealed an increased response from all studied subsets, including the iNKR^-^ subset. It is important to emphasize that this analysis did not account for all inhibitory receptors and their cognate HLA class I molecules. For example, interactions between NK cell receptor KIR2DL4 and HLA-G have recently been reported to be of importance in human breast cancer [50, 51], and neither KIR2DL4 nor HLA-G were included in our panels. Furthermore, the NK cells used in this study were derived from donors with an unknown KIR genotype, where the influence of activating KIRs could not be determined. The use of donors that only encode inhibitory KIR receptors, referred to as KIR haplotype AA donors, would have been more optimal for this type of analysis [47]. Nevertheless, the increased response observed in all subsets could imply that factors beyond reduced HLA class I expression contributes to the increased NK cell sensitivity observed in IDH2 mutated TF-1 cells.

Despite potential contributions from other mechanisms such as the above-mentioned up-regulation of activating ligands, a subgroup analysis of our boolean gating strategy indicated that the increased responsiveness was largest in an NK cell subset where the cognate HLA class I ligand was down-regulated, underscoring the importance of HLA class I in this context. Furthermore, the increased response observed against TF-1 mutant cells could be completely reverted by pre-exposing them to IFN-y, highlighting the potency of HLA class I in regulating NK cells also in this model system. Hence, our data provide strong evidence for that IDH2R140Q mutated AML cells are more sensitive to NK cells compared to IDH2WT cells, and that reduced HLA class I expression contributes to this sensitivity, but that contribution from other mechanisms, such as up-regulation of activating ligands or other today unknown factors, cannot be excluded. Besides, in a recent early phase clinical trial, where NK cells were adoptively infused to treat patients with high-risk MDS or AML, three out of six responders carried IDH2 mutations, while only one out of nine non-responders did [52]. Although this is a small study cohort, it suggests that IDH mutations indeed entails an increased susceptibility to NK cell-based immunotherapy.

The analyses performed on our AML patient cohort support a role not only of IDH2, but also IDH1 mutations in HLA down-regulation. Hence, IDH1 mutated AML patients may also benefit from NK cell-based immunotherapy. Although our model is based on the R140 mutation, the other IDH2 hotspot mutation, R172, seems to result in a higher accumulation of 2-HG [53]. Indeed, it would be interesting to more closely investigate the relationship between 2-HG levels, TET-inhibition, and NK cell sensitivity.

Finally, our results revealed that the HLA class I cluster remains hypermethylated in both mutant IDH1 and IDH2 patients resistant to IDHi. This finding is suggestive of NK cell-mediated immunotherapy as a promising target option for patients with IDH mutations, especially as a second therapeutic option in AG-221 resistant patients. The potential of NK cell-based immunotherapy is independent of the response to the IDH2i AG-221.

In addition, other IDH mutated tumor histotypes presenting the same transcriptional alterations may potentially lead to augmented susceptibly to NK cells, a particularly interesting one could be gliomas as these often carry IDH mutations [54].

Overall, this data provides new insights into the biology of IDH-mutated AML and opens up the potential for the development of new approaches for treating AML patients in a more personalized manner.

## Supplementary information

Table S1-S8

Supplemantal material

Figure S1

Figure S2

Figure S3

Figure S4

Figure S5

Figure S6

## Data Availability

The datasets have been deposited in the Gene Expression Omnibus (GEO) database (GSE207831). All data needed to evaluate the conclusions in the paper are present in the paper and/or the Supplementary Materials.
