# Supplementary material for: Perturbed epigenetic transcriptional regulation in AML with IDH mutations causes increased susceptibility to NK cells
Source: Leukemia. 2023 Jul 26;37(9):1830–41. doi: 10.1038/s41375-023-01972-3 (PMC10457197; doi:10.1038/s41375-023-01972-3)
Supplement: Supplementary file 2 — Supplemantal material [file 41375_2023_1972_MOESM2_ESM.docx]

Supplementary Information for

**Perturbed epigenetic transcriptional regulation in AML with IDH mutations causes increased susceptibility to NK cells**

Anna Palau^1^, Filip Segerberg^†,2^, Michael Lidschreiber^†,1,3^, Katja Lidschreiber^1^, Aonghus J. Naughton^1^, Maria Needhamsen^4^, Lisa Anna Jung^1^, Maja Jagodic^4^, Patrick Cramer^1,3^, Sören Lehmann^‡,*,2,6,7^, Mattias Carlsten^‡,*,2,5^, Andreas Lennartsson^‡,*,1^

*Corresponding authors. Email: andreas.lennartsson@ki.se; mattias.carlsten@ki.se; soren.lehmann@ki.se

**This PDF file includes:**

Supplementary Materials and Methods

Supplementary Figures legends (Figs. S1 to S6)

Supplementary Tables legends (Tables S1 to S8)

**Supplementary Materials and Methods**

**Cell culture**

The AML TF-1 cell lines overexpressing mutated IDH2R140Q or IDH2WT were kindly provided by Agios Pharmaceuticals^1^. The cells were cultured at 37ºC in 5% CO_2_ using RPMI 1640 medium (Gibco) supplemented with 10% fetal bovine serum (Gibco), 1% GlutaMAX (Gibco) and 2 ng/mL human GM-CSF recombinant protein (Gibco). Where indicated, the TF-1 IDH2R140Q cells were treated with 5 µM AG-221 (MedChemExpress) without GM-CSF in the medium for 4 and 7 days using DMSO as vehicle. K562 cells were obtained from ATCC (Manassas, VA, USA) and cultured in RPMI 1640 medium (Gibco) supplemented with 10% FBS at 37°C in 5% CO_2_. Where indicated, HL-60 and K562 cells were treated with 0.25 µM octyl-D-2HG (Sigma-Aldrich) for 14 days. Where indicated, TF-1 IDH2WT, IDH2R140Q and K562 cells were treated with 10 ng/mL of IFN-γ Recombinant Human Protein (Gibco) for 48 hours to stimulate HLA expression prior to NK cell co-culture experiments.

PBMCs were obtained from healthy donors in accordance with existing ethical permit (2006/229-31/3) using high-density gradient centrifugation, and viability frozen at -180°C in FBS supplemented with 10% DMSO. Upon thawing, NK cells were purified from PBMCs by magnet-assisted negative depletion using an NK cell isolation kit (Miltenyi). Before use, PBMCs and NK cells were overnight cultured in RPMI 1640 medium (Gibco) supplemented with 10% FBS (Gibco) and 1,000 IU/mL IL-2 (Peprotech) at 37°C in 5% CO_2_.

**TT-seq and RNA-seq**

TT-seq experiments were performed in biological duplicates (Spearman correlations between replicates > 0.98). A complete TT-seq step-by-step protocol can be found in the protocols.io repository^2^. Briefly, TF-1 cells were labeled with 500 μM 4-thiouridine (4sU; Sigma-Aldrich) for 15 minutes at 37°C and 5% CO2. Cells were harvested using TRIzol (ThermoFisher Scientific). Then, total RNAs were extracted according to the TRIzol manufacturer’s instructions. Total RNAs were sonicated to generate fragments of an average size < 15 knt (total fragmented RNAs) using 1.5 ml Bioruptor Plus TPX microtubes in a Bioruptor Plus instrument (Diagenode). The quality of RNAs and the size of fragmented RNAs were analyzed on a Bioanalyzer 2100 (Agilent). 1 μg of total fragmented RNAs was stored at −80°C for RNA-seq. 4sU-labeled RNAs were purified from 600 μg of total fragmented RNA. Biotinylation and purification of 4sU-labeled RNAs were performed as described^2^. Separation of 4sU-labeled RNAs was carried out with streptavidin beads (Miltenyi Biotec). Prior to library preparation, total fragmented RNAs and 4sU-labeled RNAs were treated with DNase (Qiagen), column purified (miRNeasy Micro Kit, Qiagen) and quantified using a Qubit Fluorometer (Invitrogen). The quality of RNAs was analyzed on a Bioanalyzer 2100 (Agilent). Strand-specific libraries of total fragmented RNAs (RNA-seq) and 4sU-labeled RNAs were prepared with the Ovation Universal RNA-Seq System (NuGEN/Tecan) using random hexamer priming only according to the manufacturer’s instruction with minor modifications^2^. The size selected libraries were analyzed on a Bioanalyzer 2100 (Agilent) before paired-end sequencing on the Illumina NextSeq 550. TT-Seq libraries were sequenced to the depth of ~100 million uniquely mapped reads.

**TT-seq and RNA-seq data processing**

Paired-end 75-bp reads were first mapped to a single copy of the rDNA locus to remove rRNA-related sequences. Reads that did not map to the rDNA were then aligned to the GRCh38 genome assembly (Human Genome Reference Consortium) using STAR 2.6.0c^3^ with the following specifications: outFilterMismatchNoverLmax 0.05, outFilterMultimapScoreRange 0, and alignIntronMax 500,000. Bam files were filtered with SAMtools^4^ to remove alignments with MAPQ smaller than 7 (-q 7), and only proper pairs (-f99, -f147, -f83, -f163) were selected. Fragment counts for different features were calculated with HTSeq^5^. Further data processing was carried out using the R/Bioconductor environment.

Annotation of eRNAs: Annotation of enhancer RNAs (eRNAs), was done as described^6^ with few modifications. In brief, genome-wide coverage was calculated from all TT-seq fragment midpoints in consecutive 200 bp bins throughout the genome. A two-state hidden Markov model with a Poisson-Log-Normal emission distribution was learned in order to segment the genome into ‘transcribed’ and ‘untranscribed’ states. Resulting transcribed units (TUs) were further filtered using a minimal expression threshold that was defined based on overlap with genes annotated in GENCODE (v21). The threshold was optimized using the Jaccard index criterion and resulted in ~26,000 TUs. TUs that overlapped at least 25% of an annotated protein-coding gene and overlapped with an annotated exon of the corresponding gene were classified as mRNAs. Remaining TUs were annotated as non-coding (nc)RNAs and further classified according to their genomic location relative to protein-coding genes (Figure S2F): upstream antisense RNA (uaRNA), convergent RNA (conRNA), antisense RNA (asRNA), and intergenic RNA. ncRNAs located on the opposite strand of an mRNA were classified as asRNA if the TSS was located > 1 kbp downstream of the sense TSS, as uaRNA if the TSS was located < 1 kbp upstream of the sense TSS, and as conRNA if the TSS was located < 1 kbp downstream of the sense TSS. All remaining ncRNAs were classified as intergenic. We further classified intergenic and asRNAs as eRNAs, if their TSS ± 500 bp overlapped with an enhancer state annotated by GenoSTAN^7^. In addition, we restricted our set of intergenic eRNAs to only those originating from regions with TT-seq detected transcription on both strands. The described approach was used to annotate eRNAs in TF-1 IDH2R140Q and TF-1 IDH2WT cells and annotations were subsequently merged to obtain a combined set of 4998 putative eRNAs used for all further analyses.

Identification of co-regulated enhancer-promoter pairs: Each putative enhancer that was differentially expressed between TF-1 IDH2R140Q and TF-1 IDH2WT cells (see below) was paired to a co-regulated gene’s promoter by searching for the nearest protein-coding genes (upstream, downstream and antisense) that changed expression in the same direction within a maximum distance of ± 500 kb (considering the distance between eRNA and promoter TSSs).

Differential expression analysis: Differential expression analysis of mRNAs, lncRNAs and eRNAs was performed with the DESeq2 package^8^ with the padj cutoff set to 0.05, which corresponds to a 5% false discovery rate. Gene annotations for protein-coding genes and lncRNAs were taken from RefSeq (Release 109.20190607).

**DNA methylation and hydroxymethylation analyses**

DNA methylation and hydroxymethylation assays were performed using Infinium EPIC array (Illumina) at NXT-Dx (Diagenode), requiring at least 1µg of input DNA for each sample. In brief, genomic DNA was subjected to bisulfite (BS)-treatment and oxidative BS (oxBS)-treatment using the EZ-96 DNA Methylation Kit (Zymo Research) according to Illumina’s recommended deamination protocol. Bisulfite conversion was controlled by qPCR. A quality control on the output of the Illumina Infinium EPIC array as well as the differential methylation/hydroxymethylation analyses were performed with the Bioconductor R package Chip Analysis Methylation Pipeline (ChAMP Bioconductor package version 2.18.3)^9^ using default parameters. IDAT files were taken as input files and raw β values were generated. Following initial quality check and probe filtering the data were normalized using BMIQ^10^ and limma package^11^ within ChAMP was used to calculate differential methylated probes between conditions. 5hmC β-values were calculated by subtracting BS and oxBS β-values. False positives (95^th^ percentile of the negative values) were removed. Triplicates were used except for oxBS samples at steady-state (WT and MUT) and oxBS samples at 4 days of AG-221 treatment, where duplicates were performed.

**2-HG measurement**

Supernatant from TF-1 IDH2R140Q cells treated with DMSO/AG-221 was filtered through a 10kD Spin Column (Abcam) and D-2-Hydroxyglutarate (D2HG) was measured in the filtered supernatants using the fluorometric D2HG Assay kit (Abcam) following manufacturer’s instructions.

**Phenotyping by flow cytometry**

To evaluate HLA class I surface protein expression and intensity, tumor cells were labeled with the following anti-human fluorescently-conjugated antibodies: HLA-A,B,C-PE/Cyanine7 (W6/32) and HLA-E-BV421 (3D12) from Biolegend, HLA-C-PE (DT-9) from BD Biosciences and HLA-A3-FITC (REA950), HLA-Bw4-APC (REA274) and HLA-Bw6-APC (REA143) from Miltenyi. Zombie NIR Fixable Viability kit (Biolegend) was used to discriminate between live and dead cells. All cells were acquired on an LSR II Fortessa instrument (BD Biosciences).

**Degranulation and cytokine production experiments**

Overnight IL-2 activated PBMCs were co-cultured with target cells at an effector to target (E:T) cell ratio of 10:1 for 4.5 hours at 37°C in 5% CO_2_. The co-culture with PBMCs was 1 hour in the octyl-D-2HG experiment. To measure degranulation, anti-CD107a-BV785 (LAMP-1) from Biolegend was added before the co-culture experiment was initiated. Brefeldin-A (BD Biosciences) was added after the first hour of co-culture to prevent cytokine release into the medium. At the end of the co-culture, cells were first stained with extracellular markers. Anti-CD3-V500 (UCHT1) from BD Biosciences was used to discriminate T cells. NK cells were identified by anti-CD56-BUV737 (NCAM 16.2) from BD Biosciences. The following antibodies were used to identify specific NK cell receptors and subsets: anti-KIR2DL2/2DL3/2DS2-FITC (DX27) and anti-KIR3DL1-BV421 (DX9) from Biolegend, anti-KIR2DL1/2DS1-PE (EB6) and anti-NKG2A-PECy7 (Z199) from Beckman Coulter and anti-LIR-1-APC (HP-F1) from LifeSpan Biosciences. LIVE/DEAD Fixable Aqua Dead Cell Stain Kit (Life technologies) was used to discriminate between live and dead cells. Subsequent intracellular staining was performed using the Foxp3 / Transcription Factor Staining buffer set (eBioscience) following the manufacturer´s instructions. Anti-IFN-γ-BUV395 (B27) and anti-TNF-α-BV650 (MAb11) from BD biosciences were used to detect intracellular cytokine levels. All cells were acquired on an LSR II Fortessa instrument (BD Biosciences).

**Cytotoxicity assay**

The NK cell cytotoxicity assay was performed in a similar way as to what we have previously described^12^. In brief, target cells were prelabeled with 1μM of Calcein-AM (Sigma Aldrich) for 30 min before being washed several times in RPMI 1640 medium supplemented with 10% FBS. Overnight IL-2 activated NK cells were then co-cultured with the prelabeled target cells at E:T ratios ranging from 10:1 to 0.625:1 for 4.5 hours at 37°C in 5% CO_2_. At the end of the assay, cells were thoroughly washed and resuspended in PBS, and fluorescence (535 nm) from the remaining live target cells was quantified using an Infinite M200 plate reader (TECAN). Cytotoxicity was calculated based on the linear equation generated from the cell titration ladder for each cell line.

**Data mining**

We used publicly available expression data (GEO series GSE42519) to analyze FOS gene expression in the differentiation from HSC to polymorphonuclear (PMN) granulocytes or monocytes, comprising data from isolated bone marrow cell populations of sequential differentiation stages^13^. The normalized expression values were extracted from Bloodspot database (<http://servers.binf.ku.dk/bloodspot/>).

We used publicly available DNA methylation data (GEO series GSE153347) performed using Illumina’s Infinium MethylationEPIC assay (EPIC) on patients’ samples during IDHi therapy. The differential methylation analyses were performed as stated previously in “DNA methylation and hydroxymethylation analyses” section.

**Data analysis**

Enriched gene ontology (GO) terms were extracted using GORILLA online tool^14^ and visualized with REVIGO^15^, using the following parameters: ‘Medium’ for the allowed similarity and ‘SimRel’ for semantic similarity measure.

Gene set enrichment analyses (GSEA) were performed using bioconductor R-package fgsea^16^.

Transcription factors binding sites in gene promoters or in eRNAs were conducted with HOMER Motif Analysis software from UCSD. For promoters, *findMotifs* function was used with human promoter set and default parameters. For eRNAs sequences, *findMotifsGenome* function was used with default parameters and using as input the genomic region -500bp from eRNA start to +500bp from eRNA end.

STRING network analysis was performed with version 11.5 on the web-based application at <https://string-db.org/>. Relevent protein names were inputted to “List of Names”, and “Homo sapiens” was specified as the organism in the search arguments for “Multiple proteins”. Default parameters were then used to query STRING-db to obtain a full STRING network.

Flow cytometry data analysis was performed using the FlowJo software (BD Biosciences). Microsoft Excel and GraphPad Prism was further used to generate graphs and to conduct statistical analysis.

**Supplementary materials and methods references**

1 Wang F, Travins J, Delabarre B, Penard-lacronique V, Schalm S, Hansen E *et al.* Targeted inhibition of mutant IDH2 in leukemia cells induces cellular differentiation. *Science (1979)* 2013; **340**: 622–627.

2 Gressel S, Lidschreiber K, Cramer P. Transient transcriptome sequencing: experimental protocol to monitor genome-wide RNA synthesis including enhancer transcription. *protocols.io https://doi.org/1017504/protocols.io3vzgn76* 2019.

3 Dobin A, Davis CA, Schlesinger F, Drenkow J, Zaleski C, Jha S *et al.* STAR: ultrafast universal RNA-seq aligner. *Bioinformatics* 2013; **29**: 15–21.

4 Li H, Handsaker B, Wysoker A, Fennell T, Ruan J, Homer N *et al.* The Sequence Alignment/Map format and SAMtools. *Bioinformatics* 2009; **25**: 2078–9.

5 Anders S, Pyl PT, Huber W. HTSeq--a Python framework to work with high-throughput sequencing data. *Bioinformatics* 2015; **31**: 166–9.

6 Lidschreiber K, Jung LA, Emde H, Dave K, Taipale J, Cramer P *et al.* Transcriptionally active enhancers in human cancer cells. *Mol Syst Biol* 2021; **17**: 1–23.

7 Zacher B, Michel M, Schwalb B, Cramer P, Tresch A, Gagneur J. Accurate promoter and enhancer identification in 127 ENCODE and roadmap epigenomics cell types and tissues by GenoSTAN. *PLoS One* 2017; **12**. doi:10.1371/journal.pone.0169249.

8 Love MI, Huber W, Anders S. Moderated estimation of fold change and  dispersion for RNA-seq data with DESeq2. *Genome Biol* 2014; **15**: 550.

9 Tian Y, Morris TJ, Webster AP, Yang Z, Beck S, Feber A *et al.* ChAMP: updated methylation analysis pipeline for Illumina BeadChips. *Bioinformatics* 2017; **33**: 3982–3984.

10 Teschendorff AE, Marabita F, Lechner M, Bartlett T, Tegner J, Gomez-Cabrero D *et al.* A beta-mixture quantile normalization method for correcting probe design bias in Illumina Infinium 450 k DNA methylation data. *Bioinformatics* 2013; **29**: 189–96.

11 Ritchie ME, Phipson B, Wu D, Hu Y, Law CW, Shi W *et al.* limma powers differential expression analyses for RNA-sequencing and microarray studies. *Nucleic Acids Res* 2015; **43**: e47–e47.

12 Segerberg F, Lundtoft C, Reid S, Hjorton K, Leonard D, Nordmark G *et al.* Autoantibodies to Killer Cell Immunoglobulin-Like Receptors in Patients With Systemic Lupus Erythematosus Induce Natural Killer Cell Hyporesponsiveness. *Front Immunol* 2019; **10**: 2164.

13 Rapin N, Bagger FO, Jendholm J, Mora-Jensen H, Krogh A, Kohlmann A *et al.* Comparing cancer vs normal gene expression profiles identifies new disease entities and common transcriptional programs in AML patients. *Blood* 2014; **123**: 894–904.

14 Eden E, Navon R, Steinfeld I, Lipson D, Yakhini Z. GOrilla: a tool for discovery and visualization of enriched GO terms in ranked gene lists. *BMC Bioinformatics* 2009; **10**: 48.

15 Supek F, Bošnjak M, Škunca N, Šmuc T. REVIGO summarizes and visualizes long lists of gene ontology terms. *PLoS One* 2011; **6**: e21800.

16 Korotkevich G, Sukhov V, Budin N, Shpak B, Artyomov MN, Sergushichev A. Fast gene set enrichment analysis. *biorxiv* 2019. doi:10.1101/060012.

**Supplementary figure legends**

**Fig. S1. Methylation and hydroxymethylation in TF-1 IDH2R140Q compared to TF-1 IDH2WT cells and in TF-1 IDH2R140Q mutant cells treated with AG-221. (A)** D-2-Hydroxyglutarate (D2HG) levels were measured by fluorometric assay in TF-1 IDH2WT and IDH2R140Q mutant cells condition media. **(B)** Volcano plot illustrating differentially total methylated CpG probes (adjusted p-value <0.05) comparing TF-1 IDH2R140Q versus TF-1 IDH2WT cells in BS samples (n=3). 65615 probes were found to be hypomethylated and 141406 hypermethylated in the mutant cells. **(C)** Boxplots indicating total 5hmC levels (β-value) in TF-1 IDH2WT and TF-1 IDH2R140Q mutant cells. ***, p-value <0.001. **(D)** Differentially hydroxymethylated probes (664 CpG probes with Δβ 5hmC > 0.05 and 2156 CpG probes with Δβ 5hmC < -0.05) between TF-1 IDH2R140Q mutant and TF-1 IDH2WT cells (n=2). **(E)** Genomic location in 450K enhancers, promoters or gene bodies (based on UCSC RefGene group; not annotated, 3’UTR and 5’UTR are not shown) of the 2156 hypohydroxymethylated CpG probes (Δβ 5hmC<-0.05) and the 664 hyperhydroxymethylated CpG probes (Δβ 5hmC>0.05) in TF-1 IDH2R140Q mutant vs TF-1 IDH2WT cells, comparing to location of all CpG probes in the EPIC array. Enrichment was calculated using Fisher exact t-test.*, p-value<0.01. **(F)** Genomic location in relation to CGI-related features (including CpG islands, shores, shelves and OpenSeas; not annotated are not shown) of the 2156 hypohydroxymethylated CpG probes (Δβ 5hmC<-0.05) and the 664 hyperhydroxymethylated CpG probes (Δβ 5hmC>0.05) in TF-1 IDH2R140Q mutant vs TF-1 IDH2WT cells, comparing to location of all CpG probes in the EPIC array. Enrichment was calculated using Fisher exact t-test.*, p-value<0.01. **(G)** Dotplot of 5hmC deltabeta value (Δβ-value 5hmC) of differentially hydroxymethylated CpG probes (absolute(Δβ-value 5hmC)>0.05) in relation to their 5mC deltabeta value (Δβ-value 5mC) between TF-1 IDH2R140Q and TF-1 IDH2WT cells. Pearson correlation coefficient of -0.737. **(H)** Down-regulation of the 2-HG by AG-221 treatment. TF-1 IDH2R140Q cells were treated with DMSO or AG-221 5mM for 4 days or 7 days and D-2-Hydroxyglutarate (D2HG) levels in the condition media were measured by fluorometric assay. **(I)** Boxplots indicating total 5hmC levels (β-value) in TF-1 IDH2R140Q cells treated with DMSO or AG-221 for 4 and 7 days. ***, p-value <0.001. **(J)** Boxplots indicating total 5hmC levels (beta-value) of 2156 sites which have lost 5hmC in the steady-state (MUT vs WT) in TF-1 IDH2R140Q mutant cells treated with AG-221 for 4 and 7 days. ***, p-value <0.001. **(K)** Genomic location in relation to CGI-related features (including CpG islands, shores, shelves and OpenSeas; not annotated are not shown) of the hyperhydroxymethylated CpG probes (Δβ 5hmC>0.05) in TF-1 IDH2R140Q mutant cells treated with AG-221 for 4 and 7 days, comparing to location of all CpG probes in the EPIC array. Enrichment was calculated using Fisher exact t-test.*, p-value<0.01. **(L)** Heatmap showing z-scores of Δβ 5hmC values of the 664 CpG sites which gain 5hmC in TF-1 IDH2R140Q mutant vs TF-1 IDH2WT cells in steady-state and AG-221 treatment (4 and 7 days).

**Fig. S2. TF-1 IDH2WT and IDH2R140Q cells show different expression profiles.** **(A)** Significant gene ontology (GO) terms down-regulated genes in TF-1 IDH2R140Q cells versus IDH2WT. Enriched GO terms were visualized with REVIGO, using ‘‘Medium’’ for the allowed similarity and ‘‘SimRel’’ for semantic similarity measure. **(B)** Gene set enrichment analysis (GSEA) plots comparing gene expression profiles between TF-1 IDH2R140Q and IDH2WT cells. **(C)** Differential expression analysis in long non-coding RNAs (lncRNAs). Plotted along the x-axis is the mean of log2 fold-change, along the y-axis the negative logarithm of the adjusted p-values. Red denotes the 63 up-regulated lncRNAs and the 27 down-regulated lncRNAs in the IDH2 mutant versus IDH2WT cells (p-adj value <0.05). Black denotes non-significantly differentially expressed lncRNAs. **(D)** Non-coding RNAs are classified into four categories: upstream antisense RNA (uaRNA), convergent RNA (conRNA), antisense RNA (asRNA), and intergenic RNA. asRNAs and bidirectional intergenic RNAs originating from GenoSTAN enhancer state regions^7^ are classified as putative eRNAs (orange). **(E)** DLK1 normalized eRNA counts in IDH2WT and IDH2R140 mutant (IDH2MUT) cells. The two replicates are indicated with different color.

**Fig. S3. Expression profile of TF-1 IDH2R140Q mutant cells treated with AG-221.** **(A)** Venn diagram showing the overlap of up-regulated genes at 4 and 7 days of AG-221 treatment in TF-1 IDH2R140Q cells. **(B)** Heatmap showing z-scores of Δβ 5mC values of the 171 CpG sites in promoters of the 16 genes which lose expression in TF-1 IDH2R140Q mutant vs TF-1 IDH2WT cells in steady-state and are up-regulated after AG-221 treatment (4 or 7 days). **(C)** STRING network of transcription factors with enriched binding motif activity at day 7 of AG-221 treatment in TF-1 IDH2R140Q mutant cells. Nodes in the network represent proteins and edges represent predicted associations based on various sources of evidence which include curated databases (light blue), performed experiments (purple), text-mining (yellow), co-expression (black), and homology (iris). **(D)** Gene set enrichment analysis (GSEA) plots comparing gene expression profiles between TF-1 IDH2R140Q treated with AG-221 or DMSO for 7 days. Normalized enrichment score (NES). **(E) and (F)** Significant gene ontology (GO) terms in up- and down-regulated genes in AG-221 versus DMSO treated TF-1 IDH2R140Q mutant cells after 4 days (A) or 7 days treatment (B). Enriched GO terms were visualized with REVIGO, using ‘‘Medium’’ for the allowed similarity and ‘‘SimRel’’ for semantic similarity measure.

**Fig. S4.** **Enhancer RNA transcription in TF-1 IDH2R140Q mutant cells treated with AG-221. (A)** FOS, JUN and ZEB2 normalized mRNA counts in IDH2WT and IDH2R140 mutant (IDH2MUT) cells. The two replicates are indicated with different color. **(B)** ZEB2 normalized mRNA counts in IDH2R140 mutant (IDH2MUT) cells treated with DMSO or AG-221 for 4 days. The two replicates are indicated with different color. **(C)** MYC normalized mRNA counts in IDH2WT and IDH2R140 mutant (IDH2MUT) cells. The two replicates are indicated with different color.

**Fig. S5. HLA down-regulation in IDH mutated AML. (A)** Volcano plot illustrating differentially total methylated CpG probes (adjusted p-value <0.05) comparing IDH1/2 mutated AML patients (with DNMT3AWT) (n=26) versus IDHWT (with DNMT3AWT) (n=69) in BS samples. 8871 probes were found to be hypomethylated and 110608 hypermethylated in the mutant cells. **(B)** Barplot indicating number of hypermethylated (n=46) or hypomethylated (n=0) CpG probes in HLA genes comparing IDH1/2 mutated AML patients (with DNMT3AWT) (n=26) versus IDHWT (with DNMT3AWT) (n=69) in BS samples. Genomic location of these probes in gene bodies or promoters is shown. **(C and D)** Barplots indicating number of hypermethylated (n=77) or hypomethylated (n=66) CpG probes in HLA genes comparing TF-1 IDH2R140Q and TF-1 IDH2WT cells in BS samples (n=3). Genomic location of these probes in gene bodies or promoters (C) or the class of HLA (I or II) (D) is shown.

**Fig. S6. Functional response of NK cells towards TF-1 IDH2R140Q compared to TF-1 IDH2WT cells.** **(A)** Barplots showing geometric mean fluorescence intensity (GMFI) of indicated HLA class I molecules for TF-1 IDH2WT and TF-1 IDH2R140Q cell lines from three independent staining’s (n=3). **(B)** Histograms showing representative stainings (black curves) of indicated HLA class I molecules in K562, TF-1 IDH2WT and TF-1 IDH2R140Q cell lines. Filled grey curves represent FMOs. **(C)** Zebra plots from one representative donor showing degranulation and cytokine production against no target and the denoted target cell lines. **(D)** Barplots showing degranulation and cytokine production for each denoted NK cell subset against TF-1 IDH2WT and TF-1 IDH2R140Q cell lines. The subsets were identified using a Boolean gating strategy. Bulk = all NK cells, iNKR^-^ = NK cells lacking all inhibitory receptors stained for, SP = single positive for denoted inhibitory receptor, but lacking all other inhibitory receptors stained for. Data was obtained from three independent experiments (n=12-13). **(E)** Barplots showing δ-degranulation and δ-cytokine production against TF-1 IDH2R140Q compared to TF-1 IDH2WT for denoted NK cell subsets (n=12-13). Bars in Figure D-E indicate mean. **(F)** Barplots showing geometric mean fluorescence intensity (GMFI) of indicated HLA class I molecule for TF-1 IDH2WT and TF-1 IDH2R140Q cell lines stimulated with or without 10 ng/mL of IFN-γ Recombinant Human Protein for 48 hours prior to staining. Results were obtained from two independent staining’s (n=2). **(G)** Histograms showing representative staining’s of indicated HLA class I molecules in K562, TF-1 IDH2WT and TF-1 IDH2R140Q cell lines stimulated with (dotted black curves) or without (black curves) 10 ng/mL of IFN-γ Recombinant Human Protein for 48 hours prior to staining. Filled grey curves represent FMOs. **(H)** Zebra plots from one representative donor showing degranulation and cytokine production against the denoted target cell lines stimulated with or without 10 ng/mL of IFN-γ Recombinant Human Protein for 48 hours prior to the assay. Paired or unpaired t tests were performed for all paired or unpaired analysis in this figure respectively. When no statistically significance is noted, it was either not possible to perform the test (due to low sample size), or because the result was non-significant *, p-value < 0.05; **, p-value < 0.01; ***, p-value < 0.001.

**Supplementary tables legends**

**Table S1. DESeq2 results with differentially expressed genes in TF-1 IDH2R140Q vs TF-1 IDH2WT (provided as .xls file).**

**Table S2. Enhancer-promoter pairs in TF-1 IDH2R140Q vs TF-1 IDH2WT (provided as .xls file).**

**Table S3. DESeq2 results with differentially expressed genes in TF-1 IDH2R140Q treated with AG-221 for 4 days vs TF-1 IDH2R140Q vehicle-treated (DMSO) (provided as .xls file).**

**Table S4. DESeq2 results with differentially expressed genes in TF-1 IDH2R140Q treated with AG-221 for 7 days vs TF-1 IDH2R140Q vehicle-treated (DMSO) (provided as .xls file).**

**Table S5. List of enriched motifs in down-regulated mRNAs in IDH mutated AML patients versus IDH WT AML patients’ cells (provided as .xls file).**

**Table S6. List of enriched motifs in down-regulated mRNAs in the TF-1 IDH2R140Q versus IDH2 WT cells (provided as .xls file).**

**Table S7. DESeq2 results of HLA genes in TF-1 IDH2R140Q vs TF-1 IDH2WT (provided as .xls file).**

**Table S8. DESeq2 results of HLA genes in ClinSeq AML cohort comparing IDH1/2 mutated patients vs IDH WT patients (provided as .xls file).**
