# Supplementary figures and images for: Perturbed epigenetic transcriptional regulation in AML with IDH mutations causes increased susceptibility to NK cells
Source: Leukemia. 2023 Jul 26;37(9):1830–41. doi: 10.1038/s41375-023-01972-3 (PMC10457197; doi:10.1038/s41375-023-01972-3)

**Sup Figure 1**

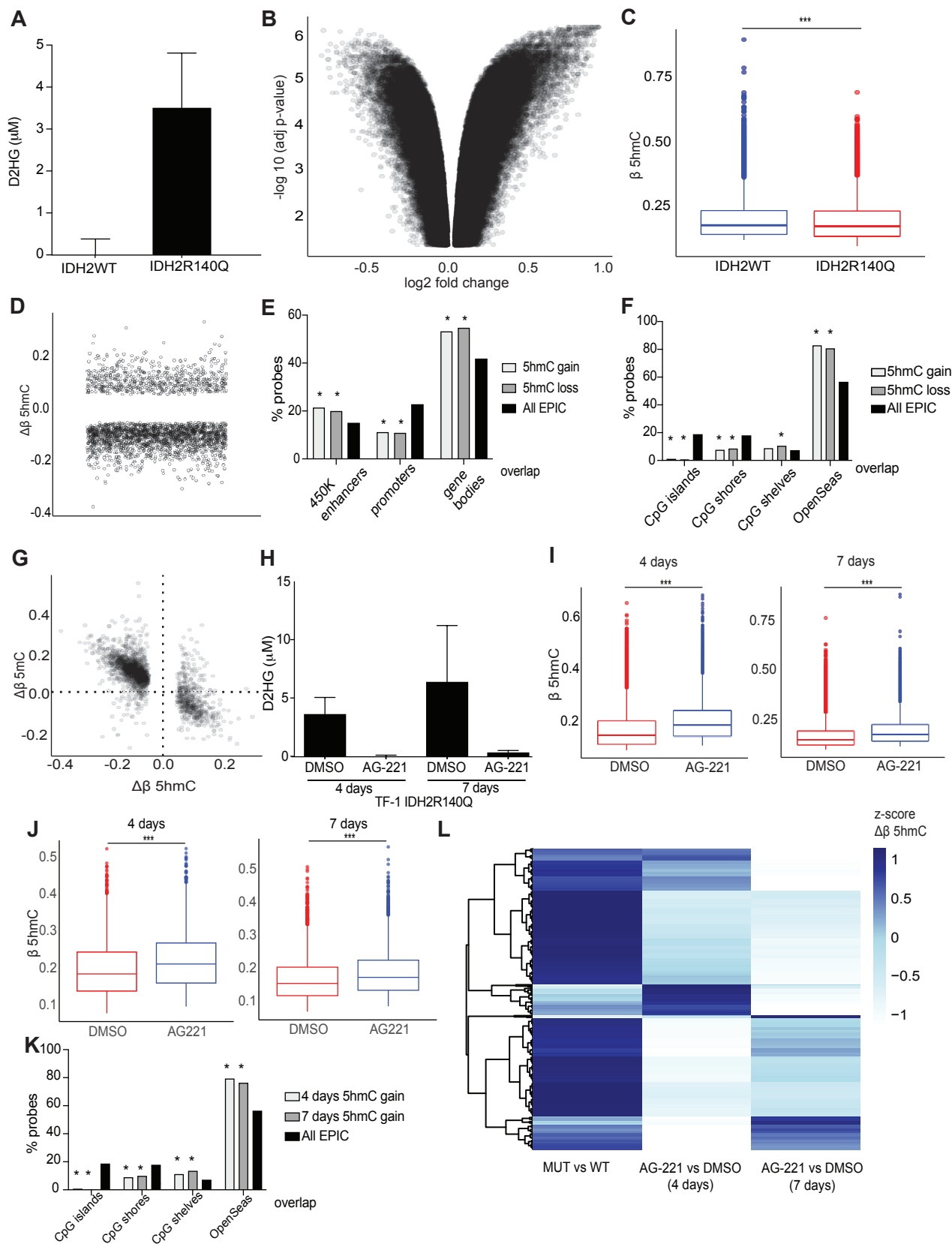

Supplement: Supplementary file 3 — Figure S1 [file 41375_2023_1972_MOESM3_ESM.pdf]

**Sup Figure 2**

**A**

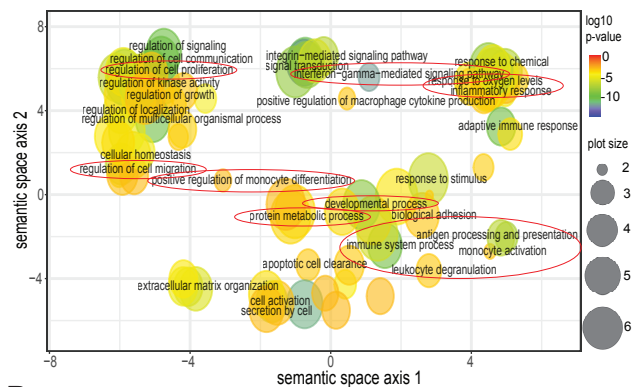

**B**

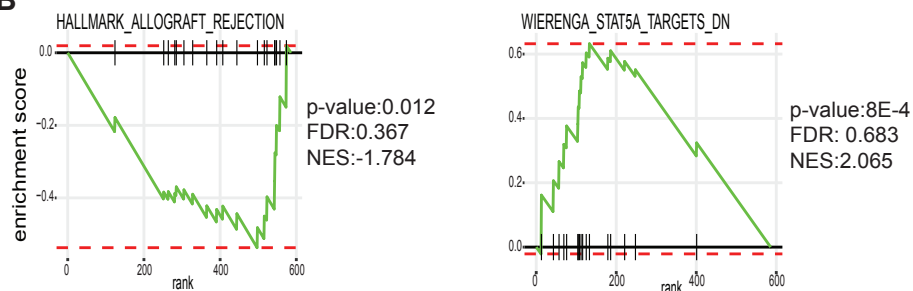

**C**

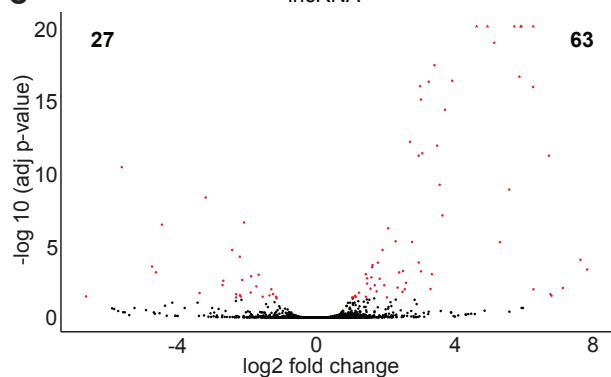

**D**

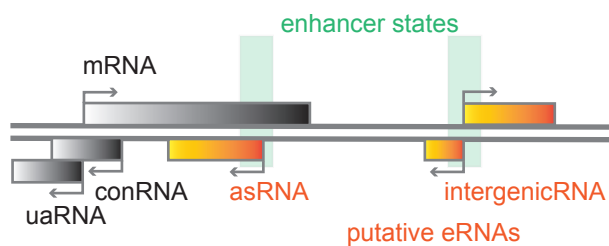

**E**

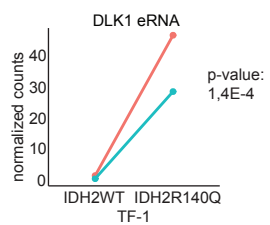

Supplement: Supplementary file 4 — Figure S2 [file 41375_2023_1972_MOESM4_ESM.pdf]

# Sup Figure 3

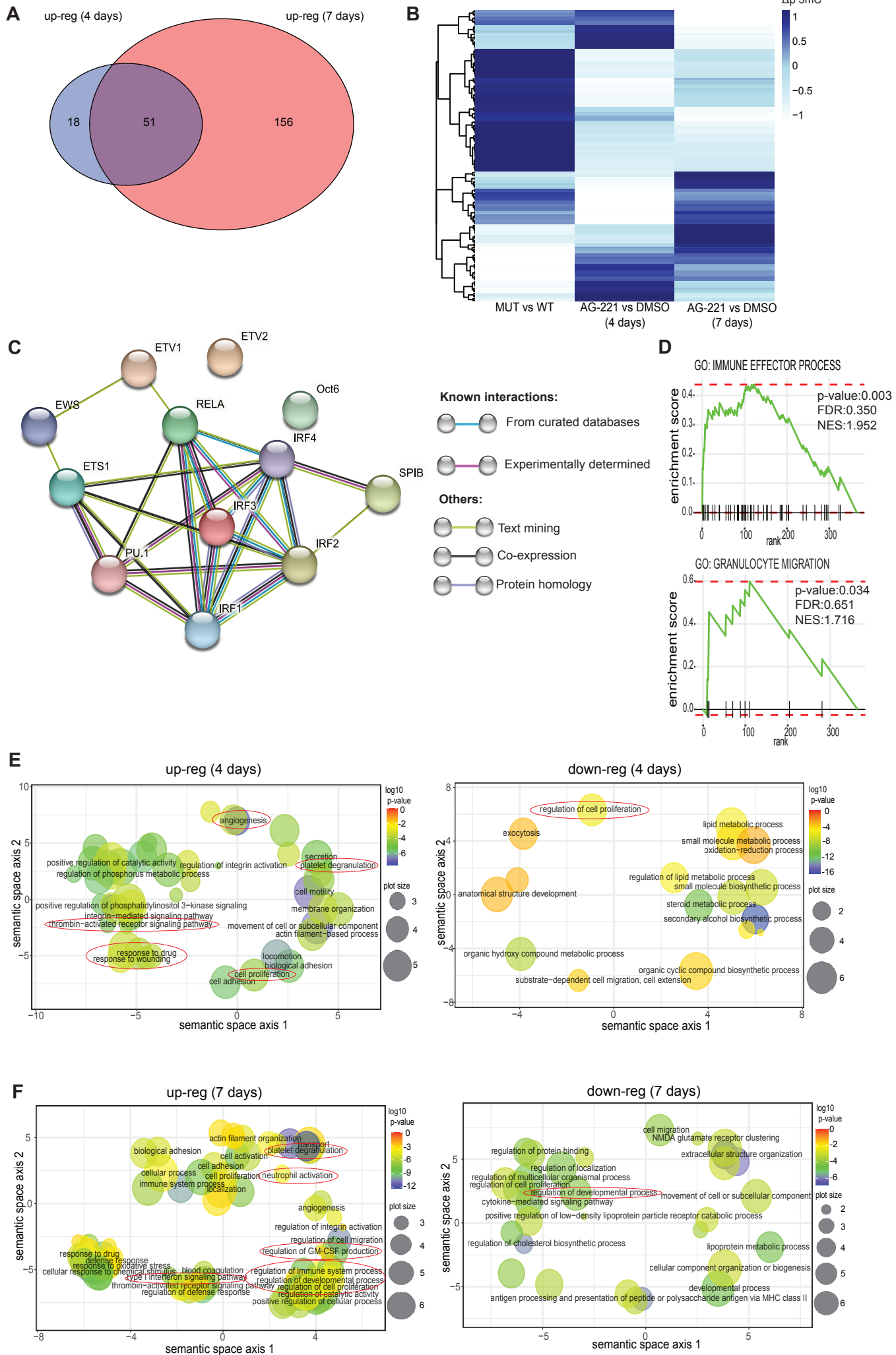

Supplement: Supplementary file 5 — Figure S3 [file 41375_2023_1972_MOESM5_ESM.pdf]

Sup Figure 4

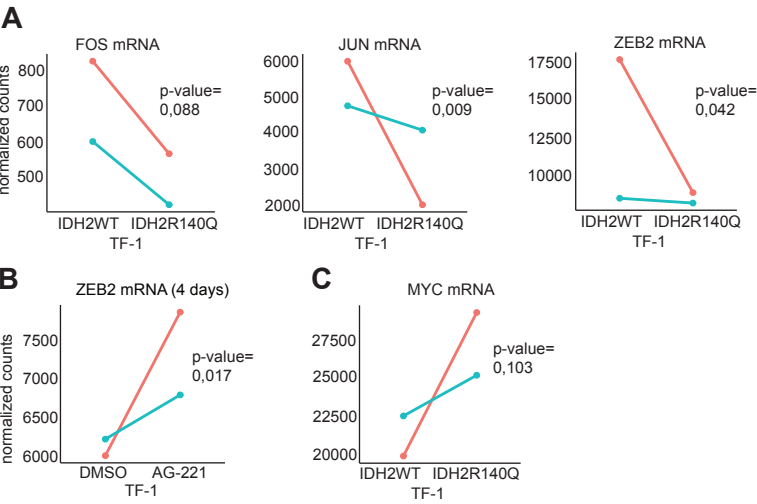

Supplement: Supplementary file 6 — Figure S4 [file 41375_2023_1972_MOESM6_ESM.pdf]

Sup Figure 5

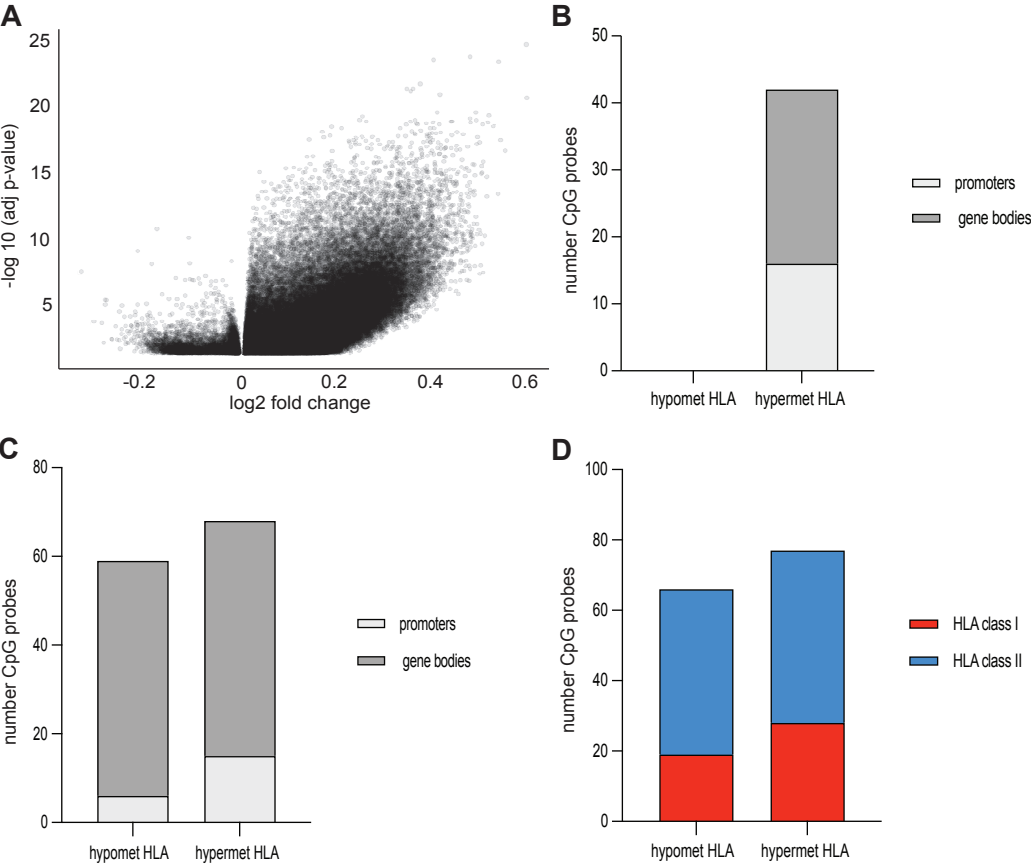

Supplement: Supplementary file 7 — Figure S5 [file 41375_2023_1972_MOESM7_ESM.pdf]

# Sup Figure 6

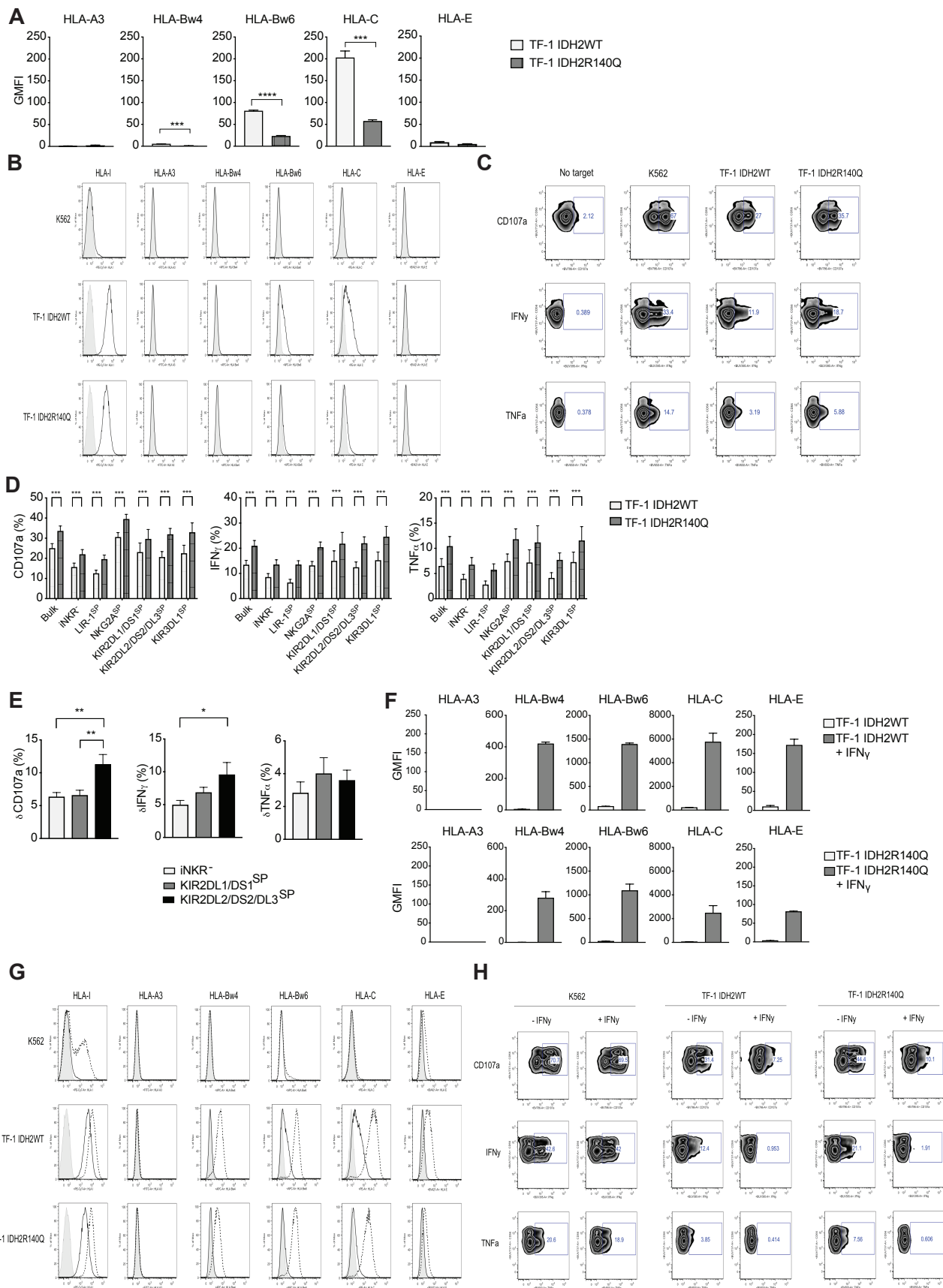

Supplement: Supplementary file 8 — Figure S6 [file 41375_2023_1972_MOESM8_ESM.pdf]
